# Mid-term follow-up after aortic valve replacement with the Carpentier Edwards Magna Ease prosthesis

**DOI:** 10.1186/s13019-020-01248-2

**Published:** 2020-08-03

**Authors:** Taufiek K. Rajab, Jason M. Ali, Jules Hernández-Sánchez, Jennifer Mackie, Vincenzo Grimaudo, Silvia Sinichino, Christine Mills, Bushra Rana, John Dunning, Yasir Abu-Omar

**Affiliations:** 1 Department of Cardiac Surgery, Papworth Hospital NHS Foundation Trust, Cambridge, UK; 2 Papworth Trial Unit Collaboration, Papworth Hospital, Cambridge, UK; 3 Biostatistics Unit, Cambridge Institute of Public Health, Cambridge, UK; 4 Edwards Lifesciences SA, Route de l’Etraz 70, 1260 Nyon, Switzerland; 5 Department of Cardiology, Papworth Hospital, Cambridge, UK

**Keywords:** Aortic stenosis, Aortic valve replacement, Carpentier magna ease, Outcomes

## Abstract

**Background:**

Approximately 250,000 heart valve operations are performed annually worldwide. An intensive research and development effort has led to progressively more advanced heart valve prostheses. The Carpentier-Edwards Perimount Magna Ease (CEPME) prosthesis represents the latest iteration of the Edwards Perimount series of aortic tissue valves. The current study aims to evaluate the midterm performance of this bioprosthesis.

**Methods:**

Five hundred and eighteen patients with aortic stenosis underwent aortic valve replacement with the CEPME valve at Papworth Hospital between August 2008 and November 2011. After a minimum of 3 years from the index operation, eligible patients were retrospectively and consecutively recruited to participate. Recruitment was closed after 100 eligible patients had completed all study assessments. Investigations at follow-up included echocardiography, and NYHA status. Primary endpoints included valve performance measures.

**Results:**

The mean age was 72 years, 64% were male and median follow-up was 5.1 years. NYHA status had improved in 66% of patients. The average postoperative peak and mean pressure gradients decreased by 51.2 mmHg (64.5%) and 31.8 mmHg (59.4%), with a significant improvement in NYHA status. The frequency of moderate aortic regurgitation was 3%. There was no evidence for structural valve deterioration.

**Conclusions:**

The CEPME has excellent mid-term durability. Its use effectively improves haemodynamics and functional capacity.

## Introduction

Aortic stenosis is the most common valvular heart disease in Europe and North America [1, 2]. Among randomly selected men and women aged 75 to 86 years, the prevalence of critical aortic valve stenosis is 2.9% [3]. Due to the aging population, approximately 150,000 patients of a total population of 64 million in England are projected to have severe aortic stenosis by 2020 [4]. Once these patients become symptomatic, their mortality rate increases to 25% per year [5, 6]. In contrast, the 10-year mortality rates among patients with symptomatic aortic stenosis who undergo aortic valve replacement (AVR) approaches the survival rate in the normal population [5, 7]. This prognostic improvement is one of the most striking achievements in modern surgery [6]. AVR is one of the most commonly performed cardiac operations, with approximately 65,000 procedures performed in in the United States per year [8, 9]. As a result, an intensive research and development effort has led to a wide variety of commercially available prostheses.

The Carpentier-Edwards Perimount series of bovine pericardial valves was originally introduced in the US in 1981 and has been continually improved since then. The latest modification of this prosthesis is the Carpentier-Edwards Perimount Magna Ease (CEPME) valve, which was introduced to the US market in 2005. The CEPME is comprised of three bovine pericardial leaflets that are preserved according to a proprietary process involving heat treatment in glutaraldehyde as well as ethanol and polysorbate 80. This process was optimized to reduce calcification of the valve leaflets after implantation. The leaflets are suspended on a flexible cobalt-chromium alloy frame. This alloy was chosen for its superior spring efficiency and fatigue-resistant characteristics. As a result, the valve is compliant at the orifice and commissures. The frame is attached to a silicone rubber sewing ring, which has been scalloped to conform to the natural anatomy of the aortic annulus. Together with the compliant nature of the frame, this facilitates coaptation between the bioprosthesis and the tissue bed. The sewing ring is covered with a porous polytetrafluoroethylene cloth that facilitates tissue ingrowth and improves biocompatibility. Finally, the profile height on the CEPME has been reduced to facilitate implantation in patients with small aortic roots.

Previously published short term outcomes with this valve have been encouraging. A series of 270 consecutive patients implanted with CEPME, showed mean transvalvular pressure gradients (MPG) of 11.8 ± 4.8 mmHg after follow-up for 150 (±91) days [10]. Another series of 99 patients implanted with CEPME showed MPG of 11.4 ± 3.1 mmHg after follow-up for 6 months [11]. Finally, a series of 132 patients implanted with CEPME showed MPG 15 ± 6 mmHg on follow-up after 2.9 ± 1.2 years [12]. However, follow-up in these studies was relatively short. The objective of this study is to analyse the mid-term results with this prosthesis.

## Methods

### Patients

A total of 518 patients underwent AVR with the CEPME prosthesis at Royal Papworth Hospital (UK) from August 2007 through to November 2011. After a minimum of 3 years since their operation, the patients were retrospectively and consecutively screened for inclusion in the study during 2015/16 when the study was performed.

Inclusion criteria were: aortic stenosis or mixed aortic valve disease, implantation of CEPME in the supra-annular position (due to this being the standard at our centre), a minimum of 3 years since valve implantation, and isolated AVR or AVR plus coronary artery bypass grafting in order to obtain a standardised patient cohort.

Exclusion criteria were participation in another clinical trial, LVEF ≤30%, prior heart surgery, bleeding diathesis, coagulopathy, renal insufficiency (creatinine ≥200 umol/L), chronic dialysis, hyperparathyroidism, alcohol abuse, echocardiographic evidence of an intracardiac mass, pre-operative myocardial infarction within 1 month, pre-operative endocarditis or sepsis within 3 months, pre-operative cerebrovascular disease within 6 months, pre-operative inotropic support within 30 days, pre-operative mechanical circulatory support within 30 days, pre-operative mechanical ventilation within 30 days, aortic dissection, emergency surgery, CEPME implantation in an intra-annular position, concomitant non-cardiac procedures, and post-operative death.

Of the 518 patients who were implanted with a Magna Ease prosthesis at Papworth between 2007 and 2011, 41 patients did not meet the inclusion criteria and 109 had died before patient screening began in 2015. Medical records were screened for the remaining 368 patients to identify eligibility. Recruitment was closed after one hundred eligible patients gave written informed consent to participate in the study, due to available funding. The study was approved by the NHS Health Research Authority Ethics Board (Trial Identifier: NCT01171625).

### Data collection

Clinical and echocardiographic data was collected at baseline preoperatively and post procedure at mid-term follow up. The following clinical data were collected: age, gender, aetiology, diagnosis, medical history, New York Heart Association (NYHA) scores, height, weight, body mass index (BMI), body surface area (BSA), date of the index procedure, type of the index procedure, and procedural information. The following echocardiographic data were collected: LV ejection fraction, aortic peak pressure gradient (PPG), aortic mean pressure gradient (MPG), effective orifice area (EOA), EOA index (EOAi) and aortic regurgitation. Finally, data on rhythm was collected from electrocardiography.

### Statistical analysis

Fisher’s exact test was used to compare NYHA scores and frequency of aortic stenosis before and after intervention. Wilcoxon’s signed rank test was used to compare pressure gradients before and after intervention. Severe aortic stenosis was defined according to the European Society of Cardiology Valvular Heart Disease guidelines as PPG > 64 mmHg or MPG > 40 mmHg [13]. The significance level was set at *p* < 0.05.

## Results

### Demographic data

A total of 100 patients were recruited to this study. The median follow-up since surgery was 5.1 years (range 3.2–7.2 years). Table 1 shows a summary of continuous variables and Table 2 a summary of discrete variables. The mean age of patients was 72 years, and 64% were male. Combined AVR and coronary artery bypass grafting was performed in 34% of patients. All patients suffered with calcific aortic valve degeneration.

**Table 1:** Baseline characteristics of continuous variables. BMI – body mass index; BSA – body surface area; EOAi – indexed effective orifice area

Variable	Mean	Median	SD	IQR	Min-Max
**Follow-up (years)**	5.2	5.1	1.1	4.2–6.1	3.1–7.1
**Age at surgery (years)**	72	72.1	7.5	66.5–78	55–85.7
**Height (cm)**	167	169	13	162–174	79–184
**Weight (kg)**	81.4	78	17.7	69–92	39–139
**BMI (kg/m**^**2**^**)**	29	27.5	6.4	25–32	19–60
**BSA (m**^**2**^**)**	1.9	1.9	0.25	1.8–2	1.3–2.6
**EOAi (cm**^**2**^**/m**^**2**^**)**	0.75	0.75	0.25	0.6–0.9	0.3–1.7

**Table 2:** Baseline characteristics of discrete variables (N.B. Total size = 100). MI: Myocardial Infarction, TIA/CVA: Transient Ischaemic Accident/Cerebrovascular Accident

Variable	Number
**Sex = Male**	64
**Pre-operative MI**	8
**Pre-operative Hypertension**	51
**Pre-operative TIA/CVA**	14
**Pre-operative heart rhythm**	
-Sinus	90
-Atrial fibrillation	9
-Paced	1
**Implanted valve size**	
-19	6
-21	21
-23	37
-25	21
-27	14
-29	1
**Heart rhythm at follow-up**	
-Sinus rhythm	73
-Atrial fibrillation	18
-Paced	9

The most frequently implanted valve sizes were 21, 23 and 25 accounting for 79% of implants. 6% of patients were implanted with a size 19. No patient in this series underwent a root enlargement procedure.

Over this period, operative mortality was 2.3%. The incidence of postoperative stroke was 2.5% and incidence of requiring a permanent pacemaker was 3.7%. The median hospital length of stay was 7.0 days (interquartile range 6.0–9.6).

### Echocardiographic results at mid-term

**Fig. 1:**
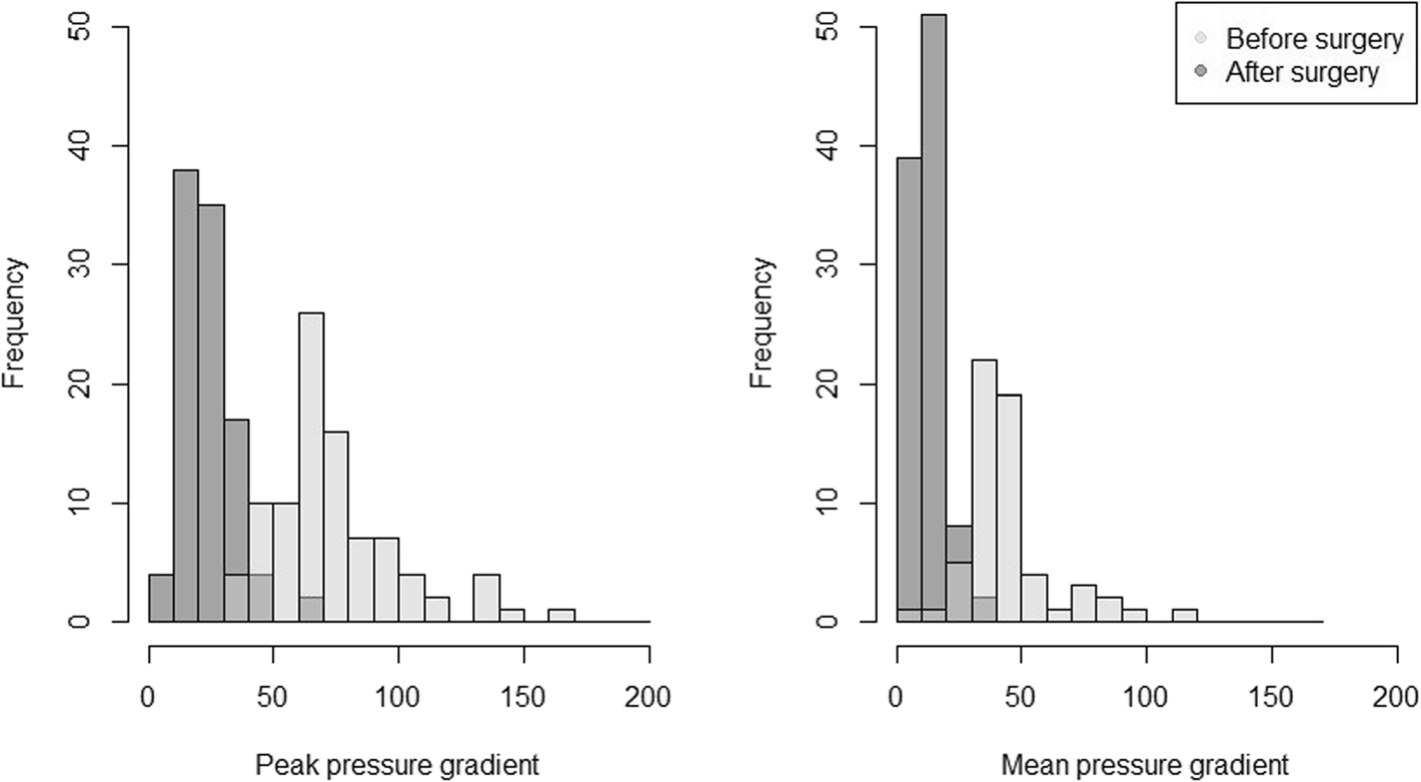
Shows aortic transvalvular pressure gradients (peak and average) at baseline and midterm follow-up after CEPME implantation

At mid-term follow-up after CEPME implantation, aortic transvalvular pressure gradients had improved significantly over baseline as expected (Fig. 1). At baseline, severe aortic stenosis was present in 88 patients according to PPG, or in 31 patients according to MPG. At mid-term follow-up after CEPME implantation, 6 patients had PPG that would fulfil the definition for severe aortic stenosis, however, when taking MPG into consideration, none of the patients fell into this severe category. Using MPG criteria, 6 patients fulfilled criteria for moderate aortic stenosis and 24 mild. Those results correspond to 94 and 100% relief of stenosis, respectively. On average, PPG had decreased by 54.2 mmHg (64.5%) (Wilcoxon’s rank test, *p* < 10^− 16^) and MPG had decreased by 31.8 mmHg (59.4%) (Wilcoxon’s rank test, p < 10^− 11^).

**Fig. 2:**
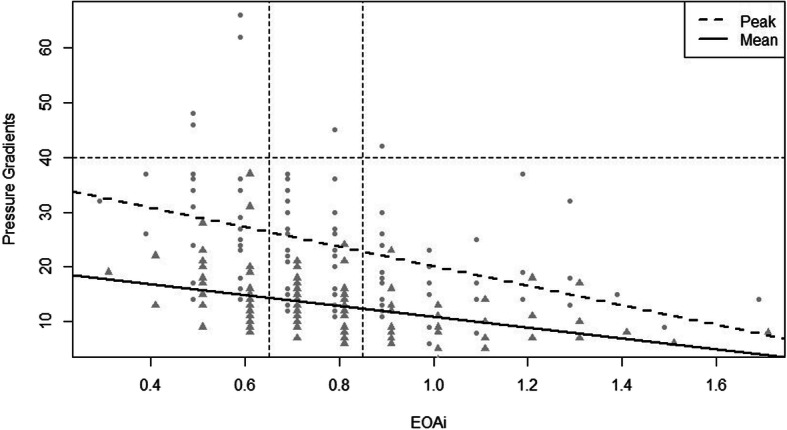
Shows simple regressions between PPG (circles) or MPG (triangles) and EOAi. The horizontal dash line in the plot separates those individuals with severe stenosis from those without. The vertical dashed lines denote thresholds for severe and moderate PPM

The pressure gradients were negatively associated with EOAi (Fig. 2). For every 0.1 unit increase in EOAi, PPG decreased by 1.8 mmHg (PPG = 37.9 (± 3.3 S.E.) – 17.7 (± 4 S.E.) EOAi, *p* = 2 × 10^− 5^, R^2^ = 16%) and MPG decreased by 1 mmHg (MPG = 20.8 (1.8 S.E.) – 9.9 (2.2 S.E.) EOAi, *p* = 1.3 × 10^− 5^, R^2^ = 17%). Only 4 patients with severe PPM had a PPG > 40 mmHg.

### NYHA

Table 3 shows the NYHA scores for categories I, II and III before and after intervention. There was a statistically highly significant NYHA classification improvement in 66% of patients after surgery (Fisher’s exact test p ~ 10^− 13^). The number of patients in the NYHA category I increased by 329% from baseline, whereas the number of patients in categories II and III decreased by 45 and 92%, respectively. There was no significant association between PPM and transvalvular gradient or NYHA status (Table 4).

**Table 3:** NYHA scores for breathlessness after physical exercise before and after intervention

NYHA	I	II	III
**Before**	21	53	26
**After**	69	29	2

**Table 4:** Change of NYHA (ΔNYHA) by PPM category

	ΔNYHA			
PPM	Better	Equal	Worse	N
**Mild**	25 (76%)	7 (21%)	1 (3%)	33
**Moderate**	25 (66%)	13 (34%)	0 (0%)	38
**Severe**	16 (55%)	8 (28%)	5 (17%)	29

### Valvular regurgitation

Thirteen percent of patients implanted with CEPME valves had mild regurgitation and 3% had moderate regurgitation on mid-term follow-up. None demonstrated severe regurgitation (Table 5). The majority of mild regurgitations were central transvalvular. Table 5 shows the number of patients with mild/moderate prosthetic valve regurgitation at mid-term follow-up by location. None of the valves in this study displayed evidence of significant structural valve deterioration. Two patients had evidence of valve leaflet thickening although this resulted in no functional compromise. Leaflet calcification was noted in one patient, again with no functional compromise.

**Table 5:** Number of patients with mild/moderate prosthetic valve regurgitation at mid-term follow-up by location

Severity	Central	Paravalvular	Undetermined	Total
**Mild**	8	2	3	13
**Moderate**	1	2	0	3

### Permanent pacemaker insertion

During the postoperative period, 8 patients required insertion of a permanent pacemaker.

## Discussion

The Carpentier-Edwards Perimount Magna Ease (CEPME) prosthesis has excellent mid-term durability, reduces transvalvular pressure gradients and improves NYHA scores. On average, peak pressure gradients and mean pressure gradients dropped by 65 and 59% after intervention. Moreover, NYHA improved in 66% of patients overall. The frequency of moderate aortic regurgitation was 3%. There was no reported structural valve deterioration. Overall significant clinical improvements were shown in this patient group after implantation.

PPM was experienced, particularly in the smaller valve sizes. Despite PPM, the majority of patients reported improvement in NYHA class and exercise tolerance. It is recognized that PPM is not an uncommon issue following AVR and has been reported with an incidence of as high as 70% [14]. A study examining the incidence of PPM following implantation of the CEPME prosthesis reported an overall incidence of severe PPM of 6.5% similar to our experience, however as expected they observed that this was strongly correlated with prosthesis size – 21.5% experienced PPM with a 19 mm prosthesis [15]. Reassuringly, it is worth noting that they concluded that there was no association between PPM and adverse clinical outcomes in the early- to mid-term – both mortality and functional status deterioration. A further study has highlighted the superiority of the CEPME valve use in patients with small aortic roots due to its larger EOA for given valve size [16].

The Edwards Perimount series of aortic bioprosthetic valves has undergone progressive design modifications to improve haemodynamic performance, durability and operative handling. The CEPME prosthesis evolved from the Perimount Magna valve. As described above, important changes include a lower profile, lower cusp height, as well as a scalloped and compliant sewing ring. While the earlier iterations in the Perimount valve series have a proven track record of excellent outcomes, only short-term data has been published for the CEPME valve and it is not known if these design modification affect the results with this valve beyond short-term follow-up [12, 17]. The current study represents the first description of mid-term results with this prosthesis, with a median duration of follow-up of over 5 years.

In addition to haemodynamic performance, durability is another key requirement for bioprostheses, especially considering that they are increasingly implanted in younger patients. Furthermore, in the era of transcatheter aortic valve implantation (TAVI), durability appears to be the area of interest when comparing outcomes. In this regard it is noteworthy a recent meta-analysis has highlighted significantly greater durability with surgical AVR prostheses compared with TAVI prostheses [18]. The CEPME prosthesis has been subject to extended in vitro study. One group subjected valve prosthesis to 1 billion cycles – equivalent to 25 years of in vivo wear and found excellent durability and hydrodynamic performance at the end of this experiment, with no episodes of valve dysfunction [19]. Although this is reassuring, in vitro experiments cannot fully replicate the in vivo environment. In this series, the CEPME prosthesis demonstrated excellent durability at mid-term follow-up. Only 3 % of the valves had moderate regurgitation and none had severe regurgitation. There was no episode of structural valve deterioration. A study reporting on mid-term outcomes of the Mitroflow bioprosthesis reported an incidence of SVD of 8.5% at only 5-years of follow-up, with a significant impact on clinical outcomes. The authors reported observing significant early calcification which they attribute to the lack of anticalcification treatment of the bioprosthesis – a feature of the CEPME prosthesis [20]. Another study has compared outcomes following the CEPME and the Trifecta valve, observing a significantly greater incidence of requiring re-intervention for SVD, highlighting the superiority of the CEPME valve prosthesis in regard to durability [21].

The study population was subjected to relatively stringent exclusion criteria to ensure a patient profile that is more representative of the average population in most centres. In spite of this, the included patients had a wide range of comorbidities. The majority of operations were isolated AVR, but one third of operations were AVR and CABG. All available CEPME valve sizes were represented in the study.

The following limitations of the current study need to be taken into account. Firstly, all patients were recruited from a single centre. Therefore, the results may reflect institutional practices and not be generalisable. Secondly, only alive patients with available data at baseline were included in the study. This is important to emphasise, as it may introduce a survival bias into the data. Similarly, patients consenting to participate may represent patients with superior outcomes, more willing to participate in the study. Thirdly, this was a retrospective study. As a result, no quality of life data at baseline was available. Fourthly, only 100 of the 518 patients with the CEPME implant were studied. This represents a low proportion and the above limitations highlight the potential bias this introduces. Unfortunately, funding limited our ability to study a greater proportion of patients. Further follow-up of this cohort is warranted to assess the longer-term durability of the CEPME bioprosthesis.

## Conclusion

The CEPME has excellent mid-term durability. Its use effectively improves haemodynamics and functional capacity.

## Data Availability

The datasets used and/or analysed during the current study are available from the corresponding author on reasonable request.

## Abbreviations

AVR: Aortic valve replacement
BMI: Body mass index
BSA: Body surface area
CEPME: Carpentier Edwards Perimount Magna Ease
EOA: Effective orifice area
MPG: Mean pressure gradient
NYHA: New York Heart Association
PPG: Peak pressure gradient
PPM: Patient prostheses mismatch
